# Unveiling Polysomal Long Non-Coding RNA Expression on the First Day of Adipogenesis and Osteogenesis in Human Adipose-Derived Stem Cells

**DOI:** 10.3390/ijms25042013

**Published:** 2024-02-07

**Authors:** Bernardo Bonilauri, Annanda Lyra Ribeiro, Lucía Spangenberg, Bruno Dallagiovanna

**Affiliations:** 1 Stem Cell Basic Biology Laboratory (LABCET), Carlos Chagas Institute—Fiocruz/PR, Curitiba 81350-010, PR, Brazil; annandalyrar@gmail.com; 2 Stanford Cardiovascular Institute, Stanford University School of Medicine, Stanford, CA 94305, USA; 3 Bioinformatics Unit, Institut Pasteur de Montevideo, Montevideo 11400, Uruguay; lucia@pasteur.edu.uy

**Keywords:** lncRNAs, adipogenesis, osteogenesis, stem cells, differentiation, polysome, translation

## Abstract

Understanding the intricate molecular mechanisms governing the fate of human adipose-derived stem cells (hASCs) is essential for elucidating the delicate balance between adipogenic and osteogenic differentiation in both healthy and pathological conditions. Long non-coding RNAs (lncRNAs) have emerged as key regulators involved in lineage commitment and differentiation of stem cells, operating at various levels of gene regulation, including transcriptional, post-transcriptional, and post-translational processes. To gain deeper insights into the role of lncRNAs’ in hASCs’ differentiation, we conducted a comprehensive analysis of the lncRNA transcriptome (RNA-seq) and translatome (polysomal-RNA-seq) during a 24 h period of adipogenesis and osteogenesis. Our findings revealed distinct expression patterns between the transcriptome and translatome during both differentiation processes, highlighting 90 lncRNAs that are exclusively regulated in the polysomal fraction. These findings underscore the significance of investigating lncRNAs associated with ribosomes, considering their unique expression patterns and potential mechanisms of action, such as translational regulation and potential coding capacity for microproteins. Additionally, we identified specific lncRNA gene expression programs associated with adipogenesis and osteogenesis during the early stages of cell differentiation. By shedding light on the expression and potential functions of these polysome-associated lncRNAs, we aim to deepen our understanding of their involvement in the regulation of adipogenic and osteogenic differentiation, ultimately paving the way for novel therapeutic strategies and insights into regenerative medicine.

## 1. Introduction

Human adipose-derived stem cells (hASCs) offer an accessible and ethically permissible source for studying stem cell biology and exploring their potential applications in cell therapy and regenerative medicine. These cells, easily isolated from adipose tissue, possess the remarkable ability to differentiate into various mesenchymal tissues such as fat (adipogenesis), bone (osteogenesis), and cartilage (chondrogenesis) [1]. Extensive research efforts have greatly contributed to our understanding of hASCs’ differentiation, shedding light on the underlying mechanisms and providing valuable insights into stem cell biology.

The differentiation process of hASCs is typically divided into two stages: lineage commitment, where stem cells transition into specific progenitor cells, and maturation, where progenitor cells differentiate into mature somatic cell types. These processes involve a complex interplay of biological factors, signaling pathways, and gene regulatory programs operating at multiple levels, including transcriptional, post-transcriptional, and post-translational regulation [2,3]. It is crucial to maintain a delicate balance between adipogenic and osteogenic differentiation, as the factors promoting adipogenesis directly inhibit osteogenesis, and vice versa. Imbalances in this equilibrium have been associated with various health conditions and diseases [4,5]. For instance, in osteoporosis patients, increased adipogenesis in the bone marrow is correlated with decreased trabecular bone volume [6]. Therefore, understanding and controlling the adipo-osteogenic fate of hASCs have significant implications in both physiological and pathological contexts.

Long non-coding RNAs (lncRNAs) have emerged as a significant class of regulatory RNA molecules involved in various biological processes, including transcription, splicing, chromatin regulation, X chromosome inactivation, telomere length regulation, cell proliferation, and the self-renewal and differentiation of stem cells. Additionally, lncRNAs have shown promise as specific biomarkers for different conditions and diseases [7,8,9,10,11]. These transcripts exert their regulatory functions both in the nucleus and in the cytoplasm, where they can form complexes with RNA-binding proteins (RBPs) and also regulate transcription, translation, and mRNA stability or degradation [12]. Notably, lncRNAs have also been found to interact with microRNAs (miRNAs) through complementary base pairing, thus acting as competitive endogenous RNAs (ceRNAs) that sequester miRNAs and prevent their binding to target mRNAs [13]. Intriguingly, despite being classified as non-coding RNAs, certain lncRNAs have been found to associate with ribosomes and polysomes, and some of them even possess small open reading frames (smORFs) that encode microproteins consisting of fewer than 100 amino acids [14,15]. This discovery has given rise to a new field of research dedicated to identifying and characterizing these smORFs within lncRNAs and investigating their potential roles as micropeptides. In light of this, given that polysomes constitute a dynamic structure facilitating the translation of RNA molecules into multiple proteins, a more thorough analysis of the association of these non-coding RNAs with such structures is imperative.

Several lncRNAs have been previously implicated in the regulation of adipogenic and osteogenic differentiation of mesenchymal stem cells, including lncRNAs associated with ribosomes [16,17]. For instance, the lncRNA *HOTAIR* has been shown to interact with Polycomb Repressive Complex 2 (PRC2), thereby regulating adipogenic-related genes crucial for lineage commitment [18]. The lncRNA *MIR99HG* has also been identified as an enhancer of adipogenesis through the targeting of miR-29b-3p and subsequent regulation of PPARγ [19]. Conversely, certain lncRNAs have been found to inhibit adipogenic differentiation, such as *GAS5*, which exerts its effects by sequestering miR-21a-5p and thereby modulating the PTEN pathway to impede adipogenesis [20]. Interestingly, *GAS5* has also been implicated as a positive regulator of osteogenic differentiation by modulating the FOXO1 and p38/JNK pathways [21,22]. Additionally, the lncRNA *H19* has been shown to exhibit increased expression in response to mechanical tension in human mesenchymal stem cells (hMSCs), promoting osteogenesis through the miR-138/FAK axis [23]. While some mechanistic insights have been proposed to elucidate lncRNA regulation during cell differentiation, further research is warranted to fully comprehend the intricate and multifaceted functions of these molecules.

In this study, we employed various bioinformatic analyses on our previously published dataset [3,24] to investigate and validate a specific lncRNA gene expression program associated with early adipogenesis and osteogenesis during cell differentiation. This lncRNA program was discernible at both the transcriptome and translatome levels, underscoring the significance of polysome-associated lncRNAs and their potential roles as competing endogenous RNAs (ceRNAs) and microprotein producers. Thus, our findings shed light on a previously concealed aspect by revealing that a majority of the identified lncRNAs are exclusively associated with polysomes.

## 2. Results

### 2.1. LncRNA Expression after 24 h of Adipogenesis and Osteogenesis Induction

**Figure 1:**
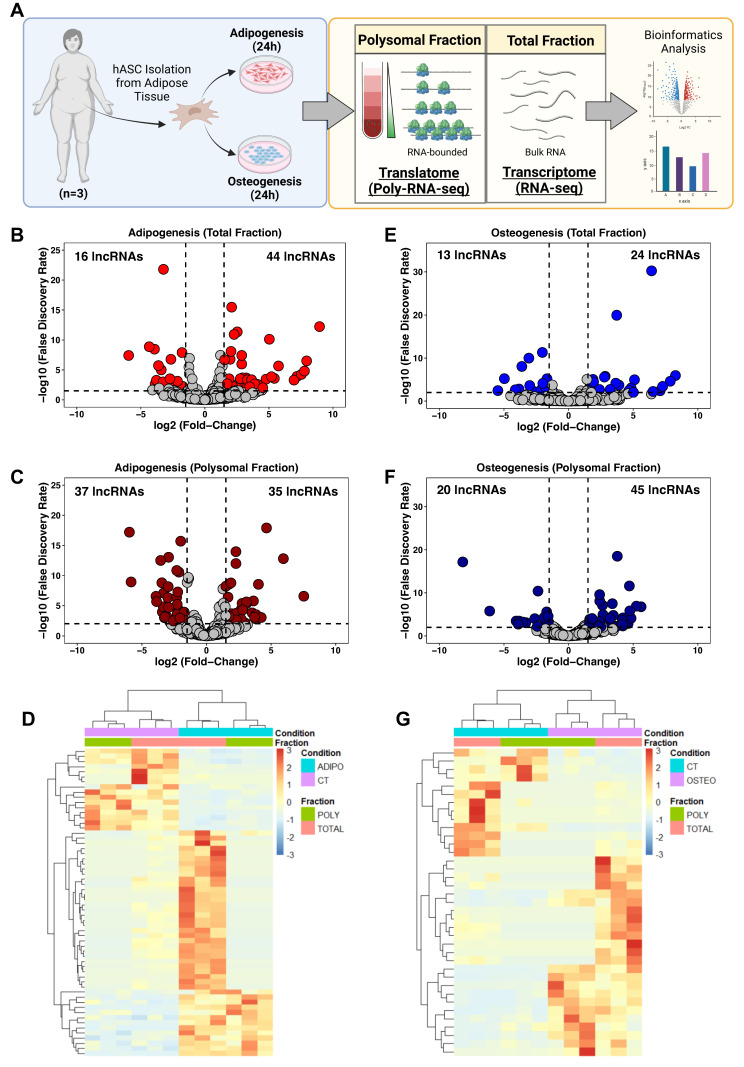
Expression profile of long non-coding RNAs following 24 h of adipogenic and osteogenic differentiation. (**A**) Schematic representation of lncRNA transcriptome and translatome of human adipose-derived stem cells (hASCs) during adipogenesis and osteogenesis. hASCs were isolated from freshly obtained subcutaneous adipose tissue of female patients (*n* = 3) after liposuction surgery. hASCs were subjected to adipogenic and osteogenic induction for 24 h, followed by polysome profiling and RNA isolation for next-generation sequencing (RNA-seq) and bioinformatics analyses. (**B**,**E**) Volcano plots depicting the differentially expressed lncRNAs in the total fraction after adipogenesis (red) and osteogenesis (blue) induction, respectively. (**C**,**F**) Volcano plots illustrating differentially expressed lncRNAs in the Polysomal fraction after adipogenesis (dark red) and osteogenesis (dark blue) induction, respectively. (**D**,**G**) Heatmap displaying the average normalized counts of differentially expressed lncRNAs in the total and polysomal fractions of undifferentiated hASCs and those undergoing adipogenesis and osteogenesis, respectively. CT: undifferentiated hASCs (control cells), ADIPO: hASCs undergoing adipogenesis, OSTEO: hASCs undergoing osteogenesis. TOTAL and POLY represent the total fraction and polysomal fraction, respectively.

To determine the expression profile of lncRNAs in hASCs during the initial stages of adipogenesis and osteogenesis differentiation, hASCs were isolated from adipose tissue obtained from patients undergoing liposuction surgery. Subsequently, adipogenic and osteogenic differentiation were triggered for 24 h followed by a polysome profiling assay and RNA isolation. We performed bulk RNA sequencing on the total (transcriptome) and polysomal (translatome) fractions, and the data were subjected to bioinformatic analysis and subsequent predictions and validations (Figure 1A).

Using our stringent cutoff criteria (see Section 4), we identified a total of 60 and 72 differentially expressed lncRNAs (DELs) after adipogenic induction in the total and polysomal fractions, respectively. Among these, 44 lncRNAs showed up-regulation and 16 lncRNAs exhibited downregulation in the total fraction, while 35 lncRNAs were upregulated and 37 lncRNAs were downregulated in the polysomal fraction (Figure 1B,C). Notably, we identified previously documented adipogenesis-related lncRNAs, including *NEAT1* [25,26] and *HOTAIR* [27] (Supplementary Tables S2 and S3).

Following 24 h of osteogenic induction, we detected 37 DELs in the total fraction and 65 DELs in the polysomal fraction. Specifically, 24 lncRNAs were upregulated and 13 lncRNAs were downregulated in the total fraction, while 45 lncRNAs were upregulated and 20 lncRNAs were downregulated in the polysomal fraction (Figure 1E,F). Additionally, we identified lncRNAs associated with osteogenic differentiation, such as *HHIP-AS1* [28] and *H19* [29] (Supplementary Tables S4 and S5). Heatmaps displaying the average expression of transcriptome and translatome DELs in undifferentiated (control) and induced conditions are presented in Figure 1D,G. Collectively, these findings demonstrate distinct expression patterns of lncRNAs in both the total and polysomal fractions following 24 h of adipogenic and osteogenic induction.

### 2.2. Characterization of DELs Related to Adipogenesis and Osteogenesis

**Figure 2:**
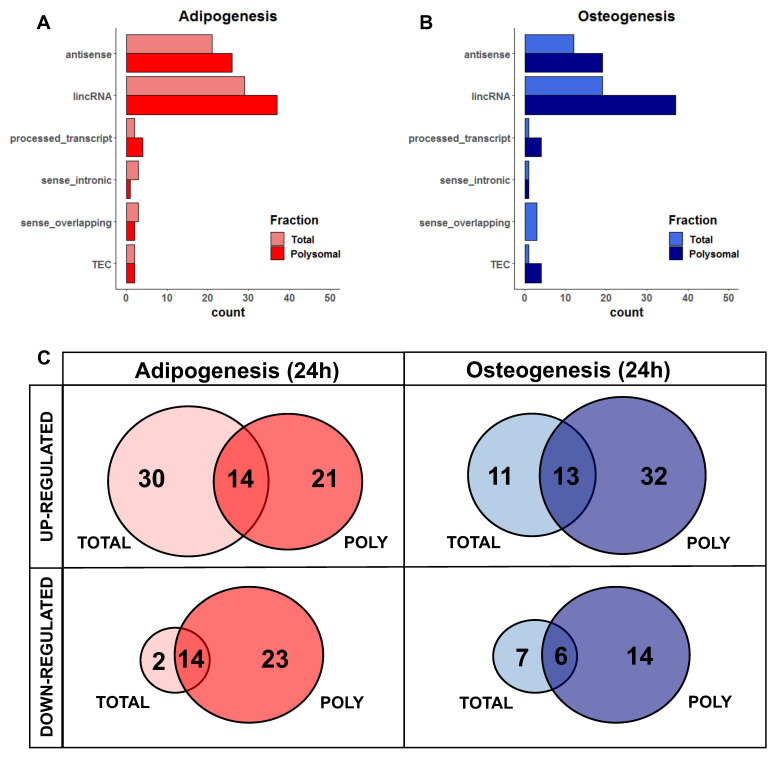
Classification of differentially expressed lncRNAs in the total and polysomal fractions after 24 h of adipogenesis and osteogenesis. (**A**,**B**) Categories of classification for adipogenic and osteogenic lncRNAs based on their genomic loci. These include lncRNAs that are antisense to protein-coding genes, intergenic lncRNAs (lincRNA) located between protein-coding genes, processed transcripts without open reading frames, lncRNAs located in intronic positions in the same sense as protein-coding genes (sense_intronic), lncRNAs overlapping exons of protein-coding genes (sense_overlapping), and transcripts awaiting experimental confirmation (TEC). (**C**) Venn diagrams illustrating the expression patterns of lncRNAs exclusively in the total fraction, exclusively in the polysomal fraction, and in both fractions following adipogenesis (represented in red) and osteogenesis (represented in blue). “TOTAL” and “POLY” refer to the total and polysomal fractions, respectively.

For a comprehensive understanding of these RNAs, we observed that the majority of DELs belong to the long intergenic non-coding RNAs (lincRNAs) class, followed by antisense lncRNAs, in both adipogenesis and osteogenesis, as well as in the total and polysomal fractions (Figure 2A,B). When considering the specificity of expression in either one (i.e., only total or only polysomal) or both fractions (i.e., common), we found that only 17.7% of upregulated DELs during the initial 24 h of adipogenesis are common between the total and polysomal fractions. Conversely, 37.9% and 26.5% of the upregulated DELs are exclusively present in the total and polysomal fractions, respectively (Figure 2C, left panel). In the case of downregulated adipogenic DELs, 26.4% are common to both the total and polysomal fractions, with only 3.77% exclusively detected in the total fraction and 43.3% exclusively detected in the polysomal fraction (Figure 2C, left panel).

Similarly, after 24 h of osteogenesis induction, we identified that 18.8% of the upregulated DELs are common to both the total and polysomal fractions. Upregulated DELs exclusively detected in the total fraction account for 15.9% of the identified transcripts, while 46.3% are exclusively detected in the polysomal fraction (Figure 2C, right panel). Regarding the downregulated osteogenic DELs, 21.2% are exclusive to the total fraction, 42.4% are exclusive to the polysomal fraction, and 18.1% are shared between both fractions (Figure 2C, right panel). Collectively, these findings shed light on the significant number of lncRNAs regulated at the polysomal level, which would be overlooked if only the total fraction was analyzed.

**Figure 3:**
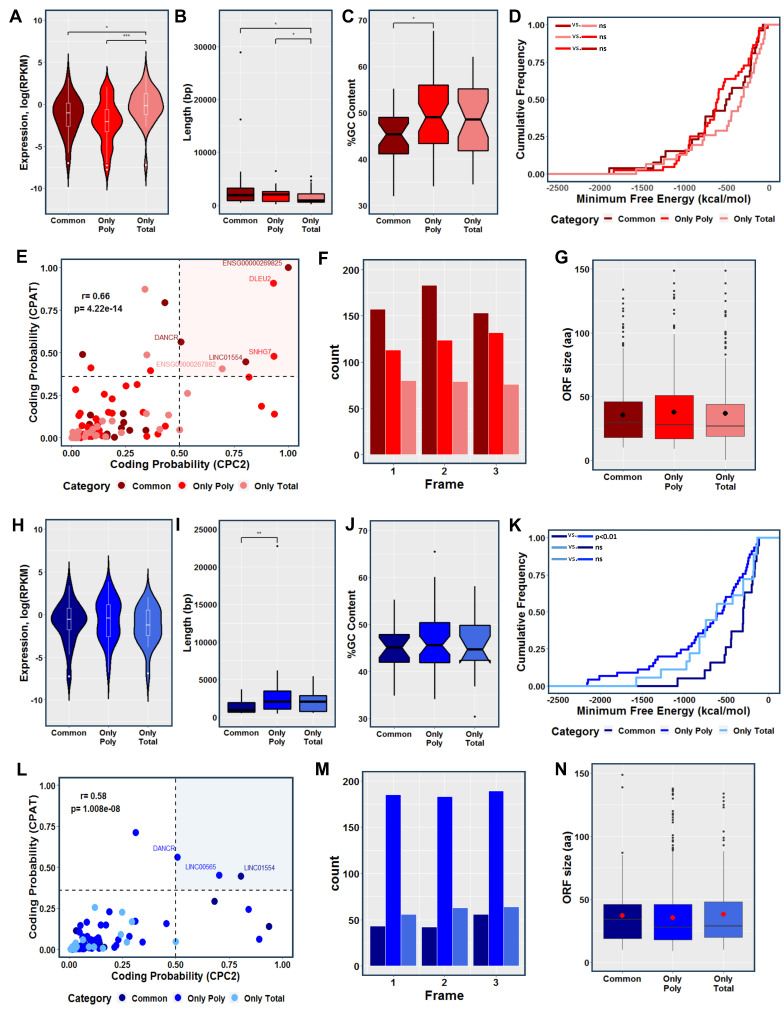
Characterization of commonly expressed, transcriptome-exclusive, and translatome-exclusive differentially expressed lncRNAs during 24 h of adipogenesis and osteogenesis. (**A**,**H**) Violin plots comparing the normalized expression levels of commonly expressed, transcriptome-exclusive, and translatome-exclusive DELs during adipogenesis and osteogenesis, respectively. (**B**,**I**) Boxplots comparing the transcript length among the three categories during adipogenesis and osteogenesis, respectively. (**C**,**J**) Comparison of the GC percentage between commonly expressed and exclusively expressed DELs during adipogenesis and osteogenesis, respectively. (**D**,**K**) Cumulative distribution of the minimum free energy (MFE) of commonly expressed, transcriptome-exclusive, and translatome-exclusive DELs during the early stages of adipogenesis and osteogenesis, respectively. (**E**,**L**) Analysis of coding probability prediction using CPC2 and CPAT for adipogenic and osteogenic DELs, respectively. (**F**,**M**) Identification of smORFs within all the identified DELs using ORFfinder during adipogenesis and osteogenesis, respectively. (**G**,**N**) Boxplots showing the average size of smORFs within adipogenic and osteogenic DELs, respectively. Common: lncRNAs expressed in both the total and polysomal fractions; Only Poly: lncRNAs exclusively expressed in the polysomal fraction; Only Total: lncRNAs exclusively expressed in the total fraction. The color red represents adipogenesis, and the color blue represents osteogenesis. Statistical significance: ns = not significant, * *p* < 0.05; ** *p* < 0.01; *** *p* < 0.001.

For a comprehensive investigation and characterization of all DELs, we further analyzed the transcripts based on their fraction-specific location (i.e., only total, only polysomal, and common). The fraction-specific lncRNAs involved in the initial stage of adipogenesis (represented in red) exhibited a similar expression pattern, characterized by a significant increase in the expression of lncRNAs exclusively detected in the transcriptome (only total fraction). Interestingly, these RNAs were significantly shorter in length compared to the common and exclusive polysome-associated lncRNAs (Figure 3A,B). In terms of GC content, the lncRNAs exclusively associated with polysomes showed a significantly higher percentage compared to the common lncRNAs (Figure 3C). However, no significant difference was observed in relation to the minimum folding energy (MFE) of these lncRNAs (Figure 3D). Using coding potential prediction analysis, we identified six lncRNAs that were classified as having encoded small open reading frames (smORFs). These included the cancer-related lncRNAs *DLEU2*, *SNHG7*, and *LINC01554* (found in only polysomal and common fractions, respectively), the recently re-annotated lncRNA ENS00000269825 (common), the novel lncRNA ENS00000267882 (only total), and the lncRNA *DANCR* (common) (Figure 3E). Examining the presence of putative coding smORFs, we observed a higher number of smORFs in the common fraction lncRNAs compared to the exclusive polysomal and total fraction lncRNAs (Figure 3F). Analyzing all the in-frame smORFs, we determined an average size of approximately 37 amino acids (aa) for the putative micropeptides across all fractions combined, 36 aa for the common lncRNAs, 37 aa for the exclusive total lncRNAs, and 39 aa for exclusively polysome-associated lncRNAs (Figure 3G and Figure S2A).

Similarly, we analyzed the fraction-specific lncRNAs involved in the initial stage of osteogenesis (represented in blue). The expression levels and GC content of these lncRNAs exhibited a similar pattern between the fractions, with a slight but significant increase in RNA length observed for the lncRNAs exclusively associated with the polysomal fraction compared to the common lncRNAs (Figure 3H,J). Notably, a significant difference in MFE was observed when comparing common and only polysomal DELs, which may be attributed to the size differences of the lncRNAs in these classes (Figure 3K). By conducting coding potential prediction analysis, we identified three lncRNAs that were classified as having encoded smORFs. These included the aforementioned lncRNA *DANCR* (only polysomal fraction) and *LINC01554* (common fraction), as well as *LINC00565* (only polysomal fraction) (Figure 3L). The presence of putative coding smORFs displayed a different pattern compared to adipogenesis-related smORFs, with a higher number of smORFs observed in the exclusive polysome-associated lncRNAs compared to the common and exclusive total lncRNAs (Figure 3M). Analyzing all the in-frame smORFs, we found an average size of approximately 39 amino acids (aa) for the putative micropeptides across all lncRNAs, 37 aa for the common lncRNAs, 42 aa for the exclusive total lncRNAs, and 39 aa for the exclusive polysome-associated lncRNAs (Figure 3G and Figure S2B). These findings highlight the lack of a consistent structural and expression pattern among fraction-specific lncRNAs after adipogenic and osteogenic induction, posing a challenge in predicting their functions and mechanisms of action.

### 2.3. Comparison between Adipogenic and Osteogenic DELs

**Figure 4:**
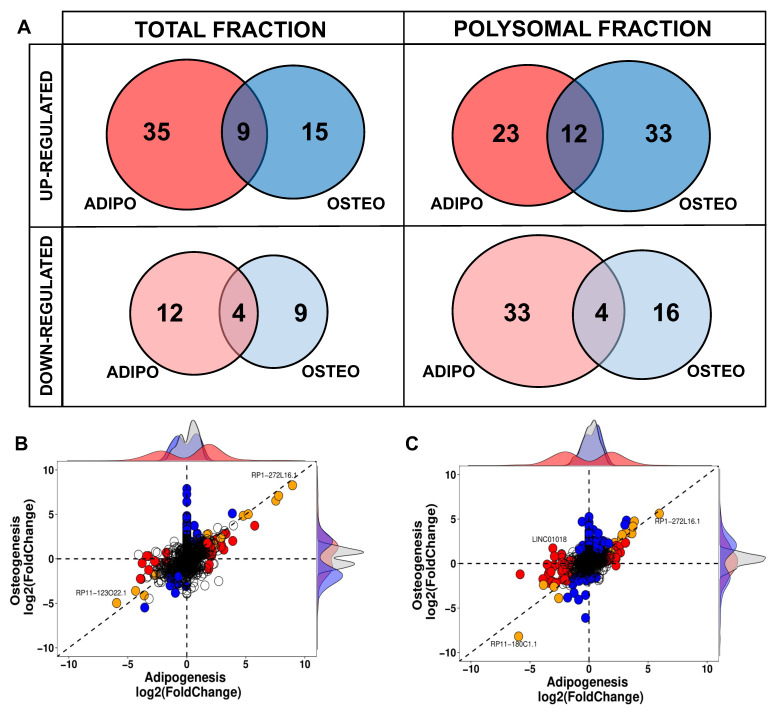
Comparison of differentially expressed lncRNAs expressed during adipogenic and osteogenic induction in total and polysomal fractions. (**A**) Venn diagrams displaying the comparison of adipogenic-related DELs (highlighted in red) and osteogenic-related DELs (highlighted in blue) in the total and polysomal fractions. (**B**,**C**) Scatter plots representing the distribution of DELs in the transcriptome and translatome, after 24 h of hASCs differentiation, respectively. Blue dots denote DELs in osteogenic differentiation, red dots signify DELs in adipogenic differentiation, and orange indicates common DELs during both osteogenesis and adipogenesis.

Another crucial characteristic of lncRNAs is their tissue-specific expression, although it remains uncertain whether this also holds true during the process of cell differentiation. In this study, we compared the DELs at the onset of adipogenesis with those expressed at the beginning of osteogenesis, and observed a distinct differentiation-specific expression pattern between the two processes. Specifically, within the first 24 h of hASCs’ differentiation, a specific gene expression program for lncRNAs associated with either adipogenesis or osteogenesis was evident in both the total and polysomal fractions (Figure 4). The small subset of commonly upregulated lncRNAs between adipogenesis and osteogenesis in the total fraction accounted for only 15.3% of all identified DELs in the transcriptome, including lncRNAs *NAV2-AS5*, *LINC00968*, and *LINC01554*. Likewise, the lncRNAs commonly upregulated in the polysomal fraction constituted only 17.6% of all identified DELs in the translatome. These included lncRNAs such as *DANCR*, *LINC00968*, and *HOXC13-AS* (Figure 4A). Conversely, the lncRNAs commonly downregulated in both differentiation processes made up only 16% of all DELs in the transcriptome. This set included lncRNAs like *RP11-123O22.1* and *APCDD1L-AS1*. In the translatome, these common downregulated lncRNAs constituted a mere 7.5% of all DELs, including lncRNAs *MYHAS* and *RP11-180C1.1* (Figure 4A). Notably, among all the identified transcripts, only the polysome-associated lncRNA *LINC01018* demonstrated up-regulation during the first 24 h of osteogenesis (log2FC: 1.71) and down-regulation during adipogenesis (log2FC: −3.06) (Figure 4C). Based on these findings, it can be concluded that the balance between adipogenic and osteogenic fate is tightly regulated in the expression of both total and polysome-associated lncRNAs, indicating a potentially important role for these lncRNAs in the determination of cell fate.

### 2.4. Polysomal LINC01018 during Early Osteogenesis and Adipogenesis

**Figure 5:**
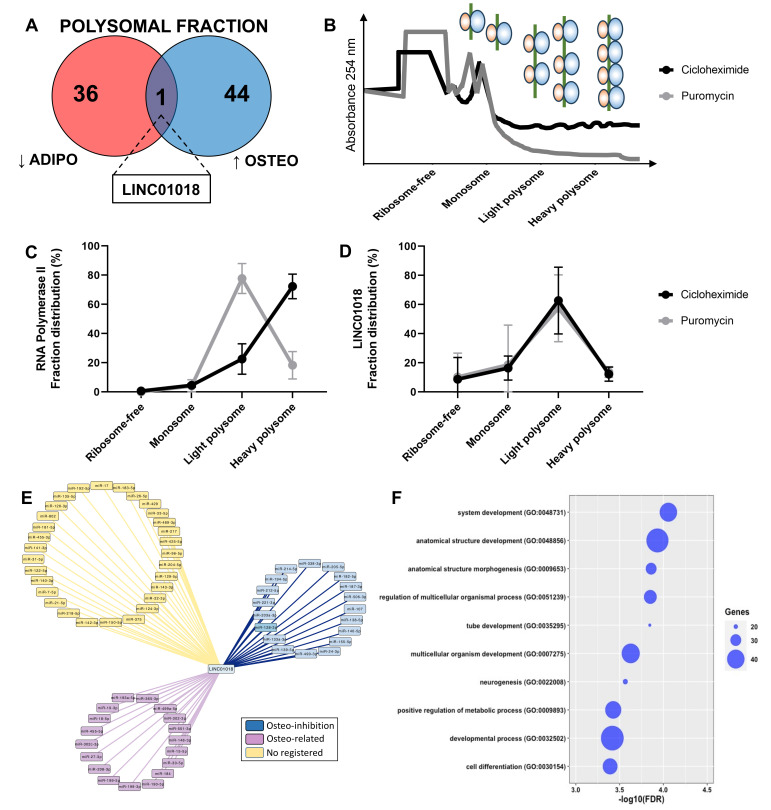
Identification and functional prediction of polysomal LINC01018. (**A**) Comparison of downregulated DELs exclusively identified in the polysomal fraction during adipogenesis and upregulated DELs exclusively identified in the polysomal fraction during osteogenesis revealed the presence of LINC01018. (**B**) Representative plot depicting the pattern of the polysome profiling assay conducted after 24 h of osteogenesis induction with and without puromycin incorporation. (**C**,**D**) Reverse transcription quantitative polymerase chain reaction (RT-qPCR) analysis demonstrating the polysomal expression of RNAP2A and LINC01018 in the presence or absence of puromycin incorporation (*n* = 3). (**E**) Predicted LINC01018-miRNA network displaying three distinct classes of miRNAs: those with documented inhibitory effects on osteogenesis (blue), those associated with osteogenesis based on publications (purple), and those without any registered publication regarding osteogenesis (yellow). (**F**) Gene ontology analysis revealing that miR-128-3p mRNA targets are involved in biological functions related to cell development, differentiation, and morphogenesis.

By comparing the upregulated and downregulated lncRNAs in both the total and polysomal fractions during adipogenic and osteogenic differentiation, we specifically pinpointed the polysome-associated lncRNA *LINC01018*. It was found to be upregulated during early osteogenesis (log2FC: 1.71) and downregulated during early adipogenesis (log2FC: −3.06) (Figure 4C and Figure 5A). To date, there is limited information available regarding the function and mechanism of action of *LINC01018* in normal cell physiology and stem cell differentiation. However, this lncRNA has been implicated in various cancer types such as glioma, colorectal cancer, hepatocellular carcinoma, and leukemia [30,31,32]. Additionally, it has been shown to regulate the in vivo expression of metabolic genes in the liver [33].

As *LINC01018* was exclusively identified as differentially expressed in the polysomal fraction through both RNA-seq and RT-qPCR (Figure 5A and Figure S3A), we conducted polysome profiling on hASCs subjected to 24 h of osteogenic induction in the presence of cycloheximide or puromycin to confirm its presence in the polysomal fraction. This analysis aimed to investigate whether *LINC01018* is directly associated with ribosomes/polysomes or co-sedimenting with other high-molecular-weight complexes such as P-bodies and complexes formed by RNA-binding proteins (RBPs) (Figure 5B). Using *POLR2A* as a control gene, we were able to observe the transition of the transcript expression from the heavy polysome to the light polysome upon puromycin incorporation (Figure 5C). In contrast, *LINC01018* was predominantly identified in the light polysomal fraction, and upon puromycin incorporation, there was no significant transition to the monosomal and free fraction, despite a slight reduction in expression in the light polysome fraction (Figure 5D and Figure S3B). This suggests that *LINC01018* may be co-sedimenting with other large complexes, rather than directly associated or translated in the polysomes.

Additionally, considering that this lncRNA may function as a competing endogenous RNA (ceRNA), we constructed a network involving potential miRNAs that have complementary sequences with *LINC01018*. We identified 67 miRNAs (Figure 5E) and categorized them based on existing literature into miRNAs associated with inhibition of osteogenesis (blue), miRNAs mentioned in any osteogenesis-related publications (purple), and miRNAs without any registered publication (yellow). Among the 19 miRNAs with documented functions as inhibitors of osteogenesis, we focused on miR-128-3p. We conducted a search for all target mRNAs of miR-128-3p among the protein-coding genes differentially expressed in the polysomal fraction during early osteogenic induction. We identified 88 upregulated mRNAs that are functionally related to system development, anatomical structure development, morphogenesis, positive metabolic processes, and cell differentiation (Figure 5F). Interestingly, a single target mRNA, *CNR1* (log2FC: 9.2), was found to be common among 7 out of the 19 miRNAs (miR-128-3p, miR-194-5p, miR-203a-3p, miR-212-5p, miR-214-5p, miR-221-3p, and miR-338-3p) known to have inhibitory effects on osteogenesis. The *CNR1* gene encodes a cannabinoid receptor that has been previously reported to be upregulated during osteogenesis and is a target of regulation by miRNAs within our network (e.g., miR-212-5p) [34]. Therefore, a potential mechanism of action for *LINC01018* may involve acting as a ceRNA that regulates the expression of *CNR1* by sequestering specific miRNAs.

Despite the potential non-translation of this lncRNA (Figure 5D), we further explored its potential to encode and produce microproteins. Using the ORFfinder program https://www.ncbi.nlm.nih.gov/orffinder/ (accessed on 2 November 2022), we identified 37 smORFs within *LINC01018*, each starting with a canonical start codon in the three reading frames. We then manually examined the two largest predicted smORFs, both 86 amino acids in length, which indicated the presence of potential microproteins located in the nucleus and cytoplasm (Figure S3E). Interestingly, when we analyzed the entire sequence of *LINC01018* using the coding potential prediction tools CPC2 [35] and RNAsamba [36], it was predicted to be a non-coding transcript (with scores of 0.07 and 0.03, respectively). However, when we specifically analyzed the selected smORFs, only smORF2 was predicted to have coding potential (with scores of 0.51 and 0.76, respectively).

## 3. Discussion

In this study, we aimed to investigate the transcriptional and translational profiles of lncRNAs during adipogenesis and osteogenesis of human adipose-derived stem cells (hASCs). Understanding the molecular mechanisms underlying the commitment of stem cells to adipocytes is crucial for gaining insights into obesity and related metabolic disorders. Mesenchymal stem cells (MSCs) have been shown to play a significant role in obesity and body weight regulation. Recent research by Yang et al. provided a comprehensive single-cell atlas of adipose tissue and skeletal muscle, highlighting the importance of MSCs in tissue adaptations associated with obesity and exercise in mice. Pathways related to extracellular matrix remodeling and circadian rhythm were found to be regulated by MSCs in response to a high-fat diet and exercise [37]. Numerous studies are currently investigating the regulatory functions of lncRNAs in obesity development, adipogenesis, and the impact of physical activity on lncRNA expression and regulation [38,39,40,41]. Additionally, Martinez et al. reported the existence of thousands of unannotated small open reading frames (smORFs) within protein-coding genes and lncRNAs in mouse adipocytes. These smORFs encode microproteins, including one with orexigenic activity in obese mice [42].

These findings underscore the importance of studying the translatome of differentiating stem cells and adipocytes to enhance our understanding of adipose tissue development and metabolic diseases like obesity, as well as to identify novel therapeutic strategies. Similar considerations apply to bone-related disorders, including osteoporosis, osteopenia, and osteonecrosis, which affect a large population worldwide [43,44].

Significant progress has been made in recent years in understanding the functions and mechanisms of lncRNAs. By manually curating the literature on the lncRNAs identified in our study, we observed that 52% of the lncRNAs in the total fraction and 65% of the lncRNAs in the polysomal fraction during early adipogenesis are discussed in at least one published scientific article, with many of them being implicated in various types of cancer. Similarly, for osteogenic differentiation, we found that 51% of the lncRNAs in both the total and polysomal fractions have related scientific articles. These findings highlight the potential importance of certain lncRNAs in the differentiation process of hASCs and in other conditions.

For instance, Chen et al. proposed a significant role for *LINC02202* (*RP11-175K6.1*) in adipogenesis of hASCs. Through a bioinformatic analysis, they demonstrated that *LINC02202* could function as a competing endogenous RNA (ceRNA), regulating PIK3R1 and FOXO1 by sequestering miR-136-5p and miR-381-3p, respectively [45]. The PI3K signaling pathway has been known to play a crucial role in adipogenic induction of stem cells [46]. In our study, we observed differential expression of *LINC02202* in both the total (log2FC: 2.5) and polysomal (log2FC: 1.58) fractions during early adipogenesis, and only in the polysomal fraction during early osteogenesis (log2FC: 2.19). Additionally, Chen et al. also reported increased expression of adipogenic-related lncRNAs, such as *SH3RF3-AS1*, *LINC01554*, *OSER1-DT*, and *LINC01914*. Consistent with their findings, our data confirm the polysomal expression of *LINC01554* and *SH3RF3-AS1*. In a recent study, Na et al. elegantly demonstrated the microprotein coding capacity of antisense *MAP3K4-AS1* (*RP3-428L16.2*). This lncRNA encoded a nuclear microprotein of 92 amino acids potentially involved in DNA replication and maintenance as it co-immunoprecipitated with MCM complex proteins (i.e., MCM2, MCM4, MCM5, and MCM7) and PARP1 [47]. However, the function of this lncRNA and microprotein during stem cell differentiation remains unknown. In our study, we observed an increased expression level of *MAP3K4-AS1* in both the total (log2FC: 2.53) and polysomal (log2FC: 2.28) fractions during early adipogenesis, suggesting enhanced translation and potential microprotein production and function.

Chen et al. demonstrated the downregulation of *LINC01119* during adipogenesis of hASCs. This lncRNA exhibited co-expression with the *PTPRB* gene, and overexpression of *PTPRB* inhibited adipogenic differentiation and reduced the expression of adipogenic-related markers [45,46]. Consistent with these findings, we also observed the downregulation of *LINC01119* in both the total (log2FC: −3.26) and polysomal (log2FC: −2.28) fractions during adipogenesis, but not during osteogenesis. Interestingly, decreased expression of *LINC01119* was also observed during osteogenic differentiation of bone-marrow mesenchymal stem cells (BMSCs) at 24 h, 48 h, and 72 h of induction. Knockdown and overexpression of *LINC01119* significantly promoted and reduced osteogenesis, respectively, along with the expression of osteogenic-related genes [48]. Collectively, these results highlight the critical role of *LINC01119* in the adipogenic and osteogenic differentiation processes of mesenchymal stem cells.

The lncRNA *ZFAS1* has been previously implicated in interactions with the ribosomal machinery and has a potential smORF that may encode a microprotein [49,50,51,52]. In our study, we observed exclusive downregulation of *ZFAS1* in the polysomal fraction (log2FC: −1.69) during osteogenic induction. Wu et al. reported a significant decrease in *ZFAS1* expression during osteogenic differentiation of BMSCs at day 1, 3, 7, and 18, while *ZFAS1* levels only appeared to increase at day 3 of adipogenic differentiation [53]. Similarly, we demonstrated the exclusive downregulation of the lncRNA *SNHG8* in the polysomal fraction (log2FC: −1.58) during osteogenic induction. Reduction of *SNHG8* expression in periodontal ligament stem cells (PDLSCs) promoted osteogenic differentiation under mechanical force, leading to a significant increase in the expression of osteogenic-related genes [54]. Recent studies have described *SNHG8* as a translated lncRNA with a coding smORF that generates a microprotein localized in the mitochondria [51,55]. However, the exact function and mechanism of action of this microprotein are still unknown.

Previously, another lncRNA named *MSC-AS1* was found to associate with ribosomes in hASCs, and was also suggested to have a smORF encoding a potential microprotein [51]. In our study, we observed downregulation of *MSC-AS1* during the early stages of osteogenesis in both the total (log2FC: −1.66) and polysomal (log2FC: −1.63) fractions. However, Zhang et al. demonstrated a significant and progressive increase in *MSC-AS1* expression during osteogenic differentiation of BMSCs, particularly at days 7 and 14 compared to day 0. They also showed that *MSC-AS1* induces osteogenesis through the miR-140-5p/BMP2/Smad axis [56]. Similarly, the expression level of the lncRNA *LINC01133* (*PAGBC*) was shown to significantly increase during osteogenic differentiation of hASCs under hydrostatic pressure. *LINC01133* acts as a sponge for miR-133b and regulates *RUNX2* expression [57]. Additionally, *LINC01133* expression progressively increased during osteogenic differentiation of human PDLSCs and acts by regulating the miR-30c/BGLAP axis [58]. In our study, we observed the downregulation of *LINC01133* in both the total (log2FC: −2.02) and polysomal (log2FC: −2.36) fractions during the early stages of osteogenesis, while no significant change was observed during adipogenesis. These findings, along with previously published data, highlight the specific temporal expression and fine-tuned program of osteogenic-related lncRNAs during the differentiation process.

### Limitations of the Study

Despite the valuable insights provided here, certain limitations warrant consideration. The use of human adipose-derived stem cells (hASCs) from subcutaneous adipose tissue obtained via liposuction surgery, while relevant for adipogenic and osteogenic studies, may limit the generalizability of findings to other stem cell types or tissue origins. Additionally, the focus on the initial 24 h of adipogenic and osteogenic induction, while capturing early transcriptional events, may not fully represent the entire differentiation process. While we employed specific techniques to validate the lncRNA *LINC01018*, further experimental validations for this and other lncRNAs are imperative. This also applies to the ceRNA hypothesis and the putative smORF encoding microproteins. Furthermore, the ceRNA network involving *LINC01018* and miRNAs presents an intriguing avenue for exploration, but requires rigorous experimental validation to confirm and elucidate their impact on osteogenesis-related genes, such as *CNR1*. Therefore, we pave the way for future comprehensive investigations into the regulatory and mechanistic roles of polysome-associated lncRNAs in adipogenic and osteogenic differentiation.

## 4. Material and Methods

### 4.1. Primary hASCs Isolation

Human adipose-derived stem cell isolation and differentiation were conducted as previously described [59]. Tissue collection after liposuction surgery were performed after donor informed consent (three biological replicates, mean age ± SD: 34.3 ± 7.13 years), in accordance with guidelines for research involving human subjects and with approval of the Ethics Committee of Oswaldo Cruz Foundation, Brazil (CAEE: 48374715.8.0000.5248). Briefly, 200 mL of fresh adipose tissue from subcutaneous fat depots were washed with phosphate-buffered saline (PBS) solution and digested with 0.1% collagenase enzyme for 30 min at 37 °C, 5% CO_2_ under constant shaking. The cell suspension was filtered and centrifuged following treatment with hemolysis buffer. The obtained cells were plated in culture flasks with DMEM supplemented with 10% FBS, penicillin (100 units/mL), and streptomycin (100 µg/mL) in an incubator at 37 °C, 5% CO_2_. Cell characterization was performed according to the International Society of Cellular Therapy guidelines [60].

### 4.2. Adipogenic and Osteogenic Differentiation of Primary hASCs

To assess the differentiation potential of freshly isolated hASCs, cells were seeded in 96-well plates (3.5 × 10^3^ or 2 × 10^3^ cells/well) and, after reaching 85% confluence, were induced to adipogenic (hMSC Adipogenic BulletKit^®^, Lonza, Basel, Switzerland) or osteogenic (hMSC Osteogenic BulletKit^®^, Lonza, Basel Switzerland) differentiation. The medium was changed every 3–4 days. Adipogenic differentiation was quantified after 21 days by examining cytoplasmic triglyceride accumulation using the AdipoRed™ Assay Reagent (Lonza, Basel Switzerland), and the formation of mineralized extracellular matrix after 28 days of osteogenic differentiation was assessed with the OsteoImage™ Mineralization Assay (Lonza, Basel Switzerland), as previously demonstrated [3,24,59]. For the polysome profiling assay, hASCs were plated in T300 culture flasks at a density of 0.2 cells/cm^2^ or 0.15 cells/cm^2^, and when reaching 85% confluence, they were treated with adipogenic (hMSC Adipogenic BulletKit^®^, Lonza) or osteogenic (hMSC Osteogenic BulletKit^®^, Lonza) medium for 24 h.

### 4.3. Polysome Profiling and RNA Purification

Polysome profiling was prepared according to a previously described procedure [59]. After 24 h of differentiation, cells were treated with 0.1 mg/mL cycloheximide (Sigma-Aldrich) or 1 mg/mL of puromycin (Sigma-Aldrich) for 60 min at 37 °C, followed by trypsinization and centrifugation. Cells were resuspended in polysome lysis buffer (15 mM Tris-HCl pH 7.4, 1% Triton X-100, 15 mM MgCl_2_, 0.3 M NaCl, 40 U RNaseOUT™, 50 U deoxyribonuclease I, and 0.1 µg/mL cycloheximide or 1 mg/mL of puromycin) and kept on ice for further 10 min. The cell lysates were centrifuged at 12,000× *g* for 10 min at 4 °C, and supernatants were loaded onto 10% to 50% sucrose gradients (BioComp model 108 Gradient Master v.5.3) and ultracentrifuged (SW40 rotor, HIMAC CP80WX HITACHI, Tokyo, Japan) at 270,000× *g* for 120 min at 4 °C. The polysome profile was recorded using the ISCO gradient fractionation system (ISCO^®^, Model 160 Gradient Former, Foxy Jr. Fraction Collector, Lincoln, NE, USA) connected to a UV detector at 254 nm absorbance. RNA from total and polysomal fractions was isolated using the Direct-zol™ RNA kit (Zymo^®^ Research, Irvine, CA, USA) according to the manufacturer’s instructions.

### 4.4. Library Construction and RNA Sequencing

The library construction and next-generation sequencing methods have been previously published [59], and the data generated were utilized for the expression analyses presented here. In summary, 1 µg of total and polysomal RNA were used for cDNA library preparation from three independent replicates of biological samples. The library was prepared using the TruSeq Stranded Total RNA Sample Preparation kit (Illumina^®^, Inc., San Diego, CA, USA) following the manufacturer’s instructions. The purified products were evaluated with an Agilent Bioanalyzer (Agilent^®^, Santa Clara, CA, USA). The high-throughput sequencing was performed in an Illumina HiSeq 2500 platform using the TruSeq SBS Kit v3—HS (Illumina^®^, Inc., San Diego, CA, USA), according to the manufacturer’s recommendations.

### 4.5. Bioinformatic Analysis

Data analysis for mapping and counting were performed with the R package Rsubread against the new version of the human genome (GRCh38). Parameters were set for unique mapping of the reads. For the determination of differential expression between undifferentiated cells (control) and differentiated cells (induced), only those genes with a count of more than 1 per million in at least three conditions were considered. For the analysis and identification of differentially expressed genes (DEGs), we used the edgeR Bioconductor package. Differentially Expressed lncRNAs (DELs) were selected using a stringent criterion using a false discovery rate (FDR) threshold of ≤1% and a log fold change (Log2FC) of ≥1.5 or ≤−1.5. All statistical and functional analyses and graphical representation were performed using R (v. 3.5.2). Principal component analysis (PCA) was verified for protein coding genes and lncRNAs (Supplementary Figure S1). The statistical analysis, including the comparison of expression levels among lncRNAs, assessment of length, and evaluation of GC content, was conducted using the Mann–Whitney U test. Significance was considered at a *p*-value of ≤0.05.

### 4.6. Prediction of lncRNA Features

The prediction of the minimum free energy (MFE) in the formation of the secondary structure of lncRNAs was calculated using RNAfold (v.2.4.14) [61]. The prediction is based on the nearest-neighbor thermodynamic model using a FASTA input file containing a lncRNA sequence from ensembl (GRCh38). The statistical comparison between the analyzed groups of lncRNAs was performed using a Mann–Whitney U test.

For identification of small open reading frames (smORFs) within lncRNAs, we used Open Reading Frame Finder (ORFfinder), https://www.ncbi.nlm.nih.gov/orffinder/ (accessed on 2 November 2022), accepting the following parameters: a minimal ORF length of 30 nucleotides (nt), and only smORFs starting with canonical ATG.

The coding potential probability was assessed using CPC2 [35] version 2.0, CPAT [62] version 3.0.4, and RNAsamba [36] version 0.2.6 software. The cutoff values for defining coding potential were set as >0.5 for CPC2, >0.3 for CPAT, and >0.5 for RNAsamba.

For protein structural and functional modeling prediction of the microproteins selected, we used the I-Tasser server [63]. Prediction of microprotein locations was performed using DeepLoc 2.0. [64]

Interaction predictions between lncRNA and microRNA were conducted using AnnoLnc2 [65]. Briefly, AnnoLnc2 computes the conservation score of interactions within the primate, mammal, and vertebrate clade. This score facilitates the identification of miRNAs with enhanced confidence, subsequently scrutinized in the literature related to osteogenesis and adipogenesis. Upon miRNA identification, all predicted miRNA-target mRNAs were retrieved from miRbase [66]. A specific miRNA was then chosen to construct a lncRNA-miRNA-mRNA network. This involved cross-referencing the anticipated target mRNAs with differentially expressed upregulated protein-coding genes during osteogenic differentiation. A gene ontology (GO) analysis was performed on these genes. The resulting network was visualized using Cytoscape (v.3.5.0).

### 4.7. cDNA Synthesis and Quantitative PCR

For both total and polysomal fractions (*n* = 3), 1 µg of RNA was used for cDNA synthesis using the Improm-II Reverse Transcription System (Promega, Madison, WI, USA), following the manufacturer’s instructions. Specifically for polysomal samples, a GFP spike was added to the cDNA reaction (internal control). Quantitative PCR was performed with GoTaq Polymerase Mix (Promega, Madison, WI, USA) and LightCycler 96 (Roche, Basel, Switzerland) equipment. Normalization was performed using the internal control POLR2A, and all reactions were performed in three technical replicates. The primers used can be checked in Supplementary Table S1.

### 4.8. Data Availability

The RNA-seq and Polysomal-RNA-seq raw data are deposited in the ArrayExpress repository under the accession number E-MTAB-6298.

## 5. Conclusions

In summary, our study utilized next-generation sequencing to comprehensively analyze the transcriptome (RNA-seq) and translatome (Poly-RNA-seq) fractions of hASCs following 24 h of adipogenic or osteogenic induction. The results obtained provide valuable insights into the expression patterns of lncRNAs, emphasizing the fraction-specific and differentiation-dependent nature of their expression. Particularly, the analysis of polysomal-associated lncRNAs highlights their regulatory potential during cellular differentiation. This aspect is of great significance, as it reveals a previously overlooked subset of lncRNAs that interact exclusively with the ribosomal machinery, potentially influencing differentiation processes through translational control and the generation of microproteins. These findings underscore the importance of considering polysomal-associated lncRNAs for a comprehensive understanding of their regulatory roles in cellular differentiation, which would have been missed by conventional analysis of total RNA fractions.

## Data Availability

Data are contained within the article and Supplementary Materials.

## Supplementary Materials

The following supporting information can be downloaded at: https://www.mdpi.com/article/10.3390/ijms25042013/s1.

## References

[1] Pittenger M.F. (1999). Multilineage Potential of Adult Human Mesenchymal Stem Cells. Science.

[2] Bonilauri B., Camillo-Andrade A.C., Santos M.D.M., de S. da G. Fischer J., Carvalho P.C., Dallagiovanna B. (2021). Proteogenomic Analysis Reveals Proteins Involved in the First Step of Adipogenesis in Human Adipose-Derived Stem Cells. Stem Cells Int..

[3] Marcon B.H., Shigunov P., Spangenberg L., Pereira I.T., de Aguiar A.M., Amorín R., Rebelatto C.K., Correa A., Dallagiovanna B. (2019). Cell Cycle Genes Are Downregulated after Adipogenic Triggering in Human Adipose Tissue-Derived Stem Cells by Regulation of mRNA Abundance. Sci. Rep..

[4] Ge C., Cawthorn W.P., Li Y., Zhao G., MacDougald O.A., Franceschi R.T. (2016). Reciprocal Control of Osteogenic and Adipogenic Differentiation by ERK/MAP Kinase Phosphorylation of Runx2 and PPARγ Transcription Factors: MAPK CONTROL OF OSTEOGENESIS AND ADIPOGENESIS. J. Cell. Physiol..

[5] Rauch A., Haakonsson A.K., Madsen J.G.S., Larsen M., Forss I., Madsen M.R., Van Hauwaert E.L., Wiwie C., Jespersen N.Z., Tencerova M. (2019). Osteogenesis Depends on Commissioning of a Network of Stem Cell Transcription Factors That Act as Repressors of Adipogenesis. Nat. Genet..

[6] Justesen J., Stenderup K., Ebbesen E.N., Mosekilde L., Steiniche T., Kassem M. (2001). Adipocyte Tissue Volume in Bone Marrow Is Increased with Aging and in Patients with Osteoporosis. Biogerontology.

[7] Mattick J.S., Amaral P.P., Carninci P., Carpenter S., Chang H.Y., Chen L.-L., Chen R., Dean C., Dinger M.E., Fitzgerald K.A. (2023). Long Non-Coding RNAs: Definitions, Functions, Challenges and Recommendations. Nat. Rev. Mol. Cell Biol..

[8] Statello L., Guo C.-J., Chen L.-L., Huarte M. (2021). Gene Regulation by Long Non-Coding RNAs and Its Biological Functions. Nat. Rev. Mol. Cell Biol..

[9] Rinn J.L., Chang H.Y. (2020). Long Noncoding RNAs: Molecular Modalities to Organismal Functions. Annu. Rev. Biochem..

[10] Bonilauri B., Dallagiovanna B. (2021). Linking Long Noncoding RNAs (lncRNAs) and Doping Detection. Drug Test. Anal..

[11] Bolha L., Ravnik-Glavač M., Glavač D. (2017). Long Noncoding RNAs as Biomarkers in Cancer. Dis. Markers.

[12] Zhao C., Xie W., Zhu H., Zhao M., Liu W., Wu Z., Wang L., Zhu B., Li S., Zhou Y. (2022). LncRNAs and Their RBPs: How to Influence the Fate of Stem Cells?. Stem Cell Res. Ther..

[13] Tay Y., Rinn J., Pandolfi P.P. (2014). The Multilayered Complexity of ceRNA Crosstalk and Competition. Nature.

[14] Chen J., Brunner A.-D., Cogan J.Z., Nuñez J.K., Fields A.P., Adamson B., Itzhak D.N., Li J.Y., Mann M., Leonetti M.D. (2020). Pervasive Functional Translation of Noncanonical Human Open Reading Frames. Science.

[15] Bonilauri B., Dallagiovanna B. (2022). Microproteins in Skeletal Muscle: Hidden Keys in Muscle Physiology. J. Cachexia Sarcopenia Muscle.

[16] Zhang P., Wu S., He Y., Li X., Zhu Y., Lin X., Chen L., Zhao Y., Niu L., Zhang S. (2022). LncRNA-Mediated Adipogenesis in Different Adipocytes. Int. J. Mol. Sci..

[17] Dallagiovanna B., Pereira I.T., Origa-Alves A.C., Shigunov P., Naya H., Spangenberg L. (2017). lncRNAs Are Associated with Polysomes during Adipose-Derived Stem Cell Differentiation. Gene.

[18] Kuo F.-C., Neville M.J., Sabaratnam R., Wesolowska-Andersen A., Phillips D., Wittemans L.B.L., van Dam A.D., Loh N.Y., Todorčević M., Denton N. (2022). HOTAIR Interacts with PRC2 Complex Regulating the Regional Preadipocyte Transcriptome and Human Fat Distribution. Cell Rep..

[19] Zhang L., Ma J., Pan X., Zhang M., Huang W., Liu Y., Yang H., Cheng Z., Zhang G., Qie M. (2022). LncRNA MIR99AHG Enhances Adipocyte Differentiation by Targeting miR-29b-3p to Upregulate PPARγ. Mol. Cell. Endocrinol..

[20] Liu H., Li H., Jin L., Li G., Hu S., Ning C., Guo J., Shuai S., Li X., Li M. (2018). Long Noncoding RNA *GAS5* Suppresses 3T3-L1 Cells Adipogenesis Through miR-21a-5p/PTEN Signal Pathway. DNA Cell Biol..

[21] Wang X., Zhao D., Zhu Y., Dong Y., Liu Y. (2019). Long Non-Coding RNA GAS5 Promotes Osteogenic Differentiation of Bone Marrow Mesenchymal Stem Cells by Regulating the miR-135a-5p/FOXO1 Pathway. Mol. Cell. Endocrinol..

[22] Yang Q., Han Y., Liu P., Huang Y., Li X., Jia L., Zheng Y., Li W. (2020). Long Noncoding RNA GAS5 Promotes Osteogenic Differentiation of Human Periodontal Ligament Stem Cells by Regulating GDF5 and P38/JNK Signaling Pathway. Front. Pharmacol..

[23] Wu J., Zhao J., Sun L., Pan Y., Wang H., Zhang W.-B. (2018). Long Non-Coding RNA H19 Mediates Mechanical Tension-Induced Osteogenesis of Bone Marrow Mesenchymal Stem Cells via FAK by Sponging miR-138. Bone.

[24] Robert A.W., Angulski A.B.B., Spangenberg L., Shigunov P., Pereira I.T., Bettes P.S.L., Naya H., Correa A., Dallagiovanna B., Stimamiglio M.A. (2018). Gene Expression Analysis of Human Adipose Tissue-Derived Stem Cells during the Initial Steps of in Vitro Osteogenesis. Sci. Rep..

[25] Cooper D.R., Carter G., Li P., Patel R., Watson J.E., Patel N.A. (2014). Long Non-Coding RNA NEAT1 Associates with SRp40 to Temporally Regulate PPARγ2 Splicing during Adipogenesis in 3T3-L1 Cells. Genes.

[26] Gernapudi R., Wolfson B., Zhang Y., Yao Y., Yang P., Asahara H., Zhou Q. (2015). miR-140 Promotes Expression of Long Non-Coding RNA NEAT1 in Adipogenesis. Mol. Cell. Biol..

[27] Divoux A., Karastergiou K., Xie H., Guo W., Perera R.J., Fried S.K., Smith S.R. (2014). Identification of a Novel lncRNA in Gluteal Adipose Tissue and Evidence for Its Positive Effect on Preadipocyte Differentiation. Obesity.

[28] Huang G., Kang Y., Huang Z., Zhang Z., Meng F., Chen W., Fu M., Liao W., Zhang Z. (2017). Identification and Characterization of Long Non-Coding RNAs in Osteogenic Differentiation of Human Adipose-Derived Stem Cells. Cell Physiol. Biochem..

[29] Li G., Yun X., Ye K., Zhao H., An J., Zhang X., Han X., Li Y., Wang S. (2020). Long Non-coding RNA-H19 Stimulates Osteogenic Differentiation of Bone Marrow Mesenchymal Stem Cells via the microRNA-149/*SDF-1* Axis. J. Cell Mol. Med..

[30] Wang S., Xu M., Sun Z., Yu X., Deng Y., Chang H. (2019). LINC01018 Confers a Novel Tumor Suppressor Role in Hepatocellular Carcinoma through Sponging microRNA-182-5p. Am. J. Physiol.-Gastrointest. Liver Physiol..

[31] Zhou H., Shi P., Jia X., Xue Q. (2021). Long Non-Coding RNA LINC01018 Inhibits the Progression of Acute Myeloid Leukemia by Targeting miR-499a-5p to Regulate PDCD4. Oncol. Lett..

[32] Su H., Hailin Z., Dongdong L., Jiang Y., Shuncheng H., Shun Z., Dan L., Biao P. (2022). Long Non-Coding RNA LINC01018 Inhibits Human Glioma Cell Proliferation and Metastasis by Directly Targeting miRNA-182-5p. J. Neurooncol.

[33] Ruan X., Li P., Chen Y., Shi Y., Pirooznia M., Seifuddin F., Suemizu H., Ohnishi Y., Yoneda N., Nishiwaki M. (2020). In Vivo Functional Analysis of Non-Conserved Human lncRNAs Associated with Cardiometabolic Traits. Nat. Commun..

[34] Chen J., Zhang Y., Liu M., Zhou Z., Li Q., Huang T., Yue Y., Tian Y. (2022). Effect and Related Mechanism of Platelet-Rich Plasma on the Osteogenic Differentiation of Human Adipose-Derived Stem Cells. BioMed Res. Int..

[35] Kang Y.-J., Yang D.-C., Kong L., Hou M., Meng Y.-Q., Wei L., Gao G. (2017). CPC2: A Fast and Accurate Coding Potential Calculator Based on Sequence Intrinsic Features. Nucleic Acids Res..

[36] Camargo A.P., Sourkov V., Pereira G.A.G., Carazzolle M.F. (2020). RNAsamba: Neural Network-Based Assessment of the Protein-Coding Potential of RNA Sequences. NAR Genom. Bioinform..

[37] Yang J., Vamvini M., Nigro P., Ho L.-L., Galani K., Alvarez M., Tanigawa Y., Renfro A., Carbone N.P., Laakso M. (2022). Single-Cell Dissection of the Obesity-Exercise Axis in Adipose-Muscle Tissues Implies a Critical Role for Mesenchymal Stem Cells. Cell Metab..

[38] Bonilauri B., Dallagiovanna B. (2020). Long Non-Coding RNAs Are Differentially Expressed after Different Exercise Training Programs. Front. Physiol..

[39] Wu B., Ding J., Chen A., Song Y., Xu C., Tian F., Zhao J. (2022). Aerobic Exercise Improves Adipogenesis in Diet-Induced Obese Mice via the lncSRA/P38/JNK/PPARγ Pathway. Nutr. Res..

[40] Chen Y.-T., Yang Q.-Y., Hu Y., Liu X.-D., de Avila J.M., Zhu M.-J., Nathanielsz P.W., Du M. (2021). Imprinted lncRNA Dio3os Preprograms Intergenerational Brown Fat Development and Obesity Resistance. Nat. Commun..

[41] Zhang F., Yang Y., Chen X., Liu Y., Hu Q., Huang B., Liu Y., Pan Y., Zhang Y., Liu D. (2021). The Long Non-Coding RNA βFaar Regulates Islet β-Cell Function and Survival during Obesity in Mice. Nat. Commun..

[42] Martinez T.F., Lyons-Abbott S., Bookout A.L., De Souza E.V., Donaldson C., Vaughan J.M., Lau C., Abramov A., Baquero A.F., Baquero K. (2023). Profiling Mouse Brown and White Adipocytes to Identify Metabolically Relevant Small ORFs and Functional Microproteins. Cell Metab..

[43] Sozen T., Ozisik L., Calik Basaran N. (2017). An Overview and Management of Osteoporosis. Eur. J. Rheumatol..

[44] Patil S., Dang K., Zhao X., Gao Y., Qian A. (2020). Role of LncRNAs and CircRNAs in Bone Metabolism and Osteoporosis. Front. Genet..

[45] Chen K., Xie S., Jin W. (2019). Crucial lncRNAs Associated with Adipocyte Differentiation from Human Adipose-Derived Stem Cells Based on Co-Expression and ceRNA Network Analyses. PeerJ.

[46] Kim J., Han D., Byun S.-H., Kwon M., Cho S.-J., Koh Y.H., Yoon K. (2017). Neprilysin Facilitates Adipogenesis through Potentiation of the Phosphatidylinositol 3-Kinase (PI3K) Signaling Pathway. Mol. Cell Biochem..

[47] Na Z., Dai X., Zheng S.-J., Bryant C.J., Loh K.H., Su H., Luo Y., Buhagiar A.F., Cao X., Baserga S.J. (2022). Mapping Subcellular Localizations of Unannotated Microproteins and Alternative Proteins with MicroID. Mol. Cell.

[48] Gao H., Dong H., Zheng J., Jiang X., Gong M., Hu L., He J., Wang Y. (2022). LINC01119 Negatively Regulates Osteogenic Differentiation of Mesenchymal Stem Cells via the Wnt Pathway by Targeting FZD4. Stem Cell Res. Ther..

[49] Hansji H., Leung E.Y., Baguley B.C., Finlay G.J., Cameron-Smith D., Figueiredo V.C., Askarian-Amiri M.E. (2016). ZFAS1: A Long Noncoding RNA Associated with Ribosomes in Breast Cancer Cells. Biol. Direct.

[50] Razooky B., Obermayer B., O’May J., Tarakhovsky A. (2017). Viral Infection Identifies Micropeptides Differentially Regulated in smORF-Containing lncRNAs. Genes.

[51] Bonilauri B., Holetz F.B., Dallagiovanna B. (2021). Long Non-Coding RNAs Associated with Ribosomes in Human Adipose-Derived Stem Cells: From RNAs to Microproteins. Biomolecules.

[52] Guo Z.-W., Meng Y., Zhai X.-M., Xie C., Zhao N., Li M., Zhou C.-L., Li K., Liu T.-C., Yang X.-X. (2019). Translated Long Non-Coding Ribonucleic Acid ZFAS1 Promotes Cancer Cell Migration by Elevating Reactive Oxygen Species Production in Hepatocellular Carcinoma. Front. Genet..

[53] Wu J., Lin T., Gao Y., Li X., Yang C., Zhang K., Wang C., Zhou X. (2022). Long Noncoding RNA ZFAS1 Suppresses Osteogenic Differentiation of Bone Marrow-Derived Mesenchymal Stem Cells by Upregulating miR-499-EPHA5 Axis. Mol. Cell. Endocrinol..

[54] Zhang Z., He Q., Yang S., Zhao X., Li X., Wei F. (2022). Mechanical Force-Sensitive lncRNA SNHG8 Inhibits Osteogenic Differentiation by Regulating EZH2 in hPDLSCs. Cell. Signal..

[55] van Heesch S., Witte F., Schneider-Lunitz V., Schulz J.F., Adami E., Faber A.B., Kirchner M., Maatz H., Blachut S., Sandmann C.-L. (2019). The Translational Landscape of the Human Heart. Cell.

[56] Zhang N., Hu X., He S., Ding W., Wang F., Zhao Y., Huang Z. (2019). LncRNA MSC-AS1 Promotes Osteogenic Differentiation and Alleviates Osteoporosis through Sponging microRNA-140–5p to Upregulate BMP2. Biochem. Biophys. Res. Commun..

[57] Ru J., Guo L., Ji Y., Niu Y. (2020). Hydrostatic Pressure Induces Osteogenic Differentiation of Adipose-Derived Mesenchymal Stem Cells through Increasing lncRNA-PAGBC. Aging.

[58] Li Q., Zhou H., Wang C., Zhu Z. (2022). Long non-coding RNA Linc01133 promotes osteogenic differentiation of human periodontal ligament stem cells via microRNA-30c / bone gamma-carboxyglutamate protein axis. Bioengineered.

[59] Marcon B.H., Spangenberg L., Bonilauri B., Robert A.W., Angulski A.B.B., Cabo G.C., Cofré A.R., Bettes P.S.L., Dallagiovanna B., Shigunov P. (2020). Data Describing the Experimental Design and Quality Control of RNA-Seq of Human Adipose-Derived Stem Cells Undergoing Early Adipogenesis and Osteogenesis. Data Brief.

[60] Dominici M., Le Blanc K., Mueller I., Slaper-Cortenbach I., Marini F., Krause D., Deans R., Keating A., Prockop D., Horwitz E. (2006). Minimal Criteria for Defining Multipotent Mesenchymal Stromal Cells. The International Society for Cellular Therapy Position Statement. Cytotherapy.

[61] Lorenz R., Bernhart S.H., Höner zu Siederdissen C., Tafer H., Flamm C., Stadler P.F., Hofacker I.L. (2011). ViennaRNA Package 2.0. Algorithms Mol. Biol..

[62] Wang L., Park H.J., Dasari S., Wang S., Kocher J.-P., Li W. (2013). CPAT: Coding-Potential Assessment Tool Using an Alignment-Free Logistic Regression Model. Nucleic Acids Res..

[63] Yang J., Yan R., Roy A., Xu D., Poisson J., Zhang Y. (2015). The I-TASSER Suite: Protein Structure and Function Prediction. Nat. Methods.

[64] Thumuluri V., Almagro Armenteros J.J., Johansen A.R., Nielsen H., Winther O. (2022). DeepLoc 2.0: Multi-Label Subcellular Localization Prediction Using Protein Language Models. Nucleic Acids Res..

[65] Ke L., Yang D.-C., Wang Y., Ding Y., Gao G. (2020). AnnoLnc2: The One-Stop Portal to Systematically Annotate Novel lncRNAs for Human and Mouse. Nucleic Acids Res..

[66] Kozomara A., Griffiths-Jones S. (2011). miRBase: Integrating microRNA Annotation and Deep-Sequencing Data. Nucleic Acids Res..

