# Supplementary material for: Unveiling Polysomal Long Non-Coding RNA Expression on the First Day of Adipogenesis and Osteogenesis in Human Adipose-Derived Stem Cells
Source: Int J Mol Sci. 2024 Feb 7;25(4):2013. doi: 10.3390/ijms25042013 (PMC10888724; doi:10.3390/ijms25042013)
Supplement: Supplementary file 1 [file ijms-25-02013-s001.zip › ijms-2794617-supplementary.pdf]

SUPPLEMENTARY FILES

For

Unveiling polysomal long non-coding RNA expression during adipogenesis and osteogenesis of human adipose-derived stem cells

Bernardo Bonilauri[1,2]*, Annanda Lyra Ribeiro[1], Lucia Spangenberg[3], Bruno Dallagiovanna[1]*

Supplementary Figure 1

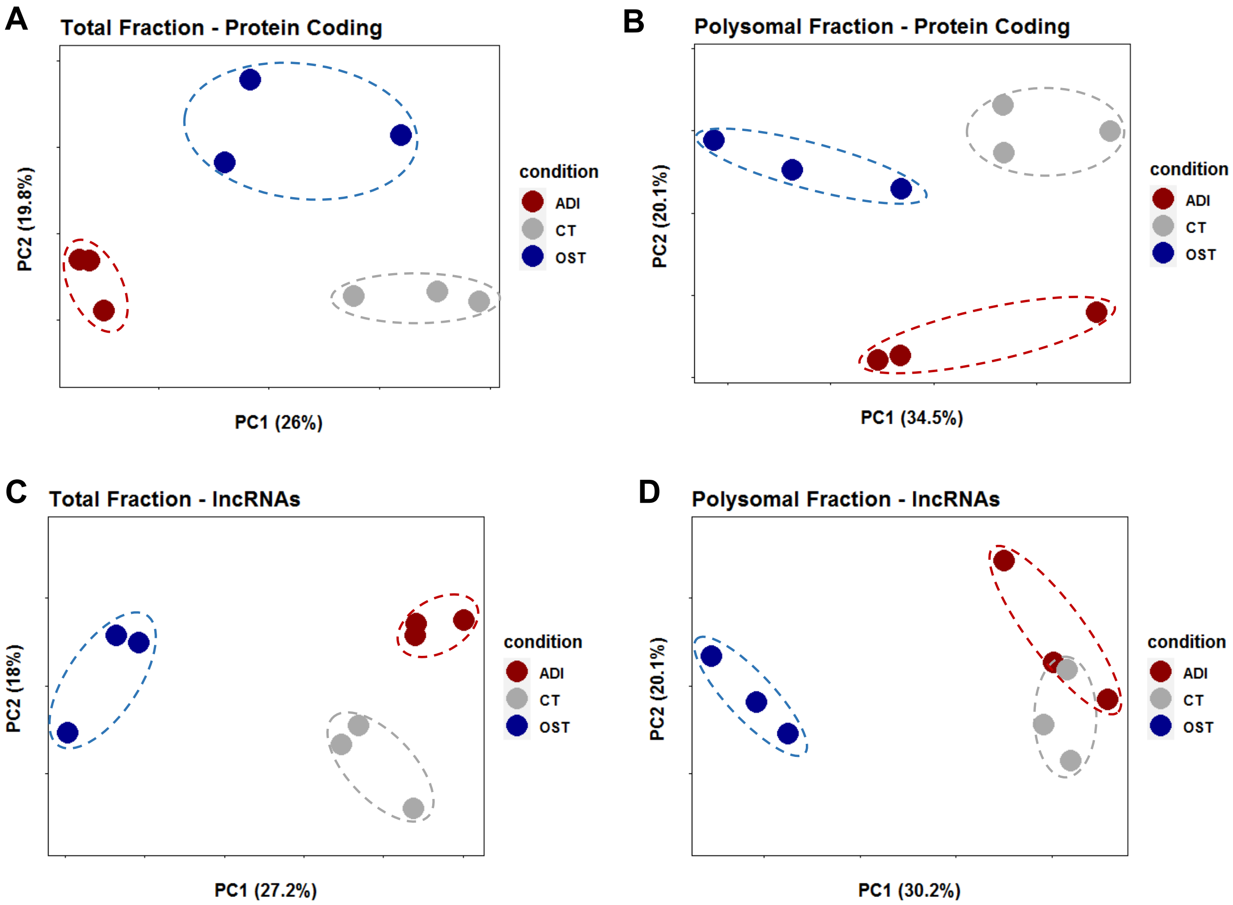

Figure S1: Principal Component Analysis (PCA) of normalized data. (A and C) PCA plot showing the distribution of mRNAs and lncRNAs in the total fraction, respectively. **(B and D)** PCA plot depicting the distribution of mRNAs and lncRNAs in the polysomal fraction, respectively. The conditions are represented as ADI for adipogenesis (red), OST for osteogenesis (blue), and CT for control-undifferentiated cells (gray).

Supplementary Figure 2

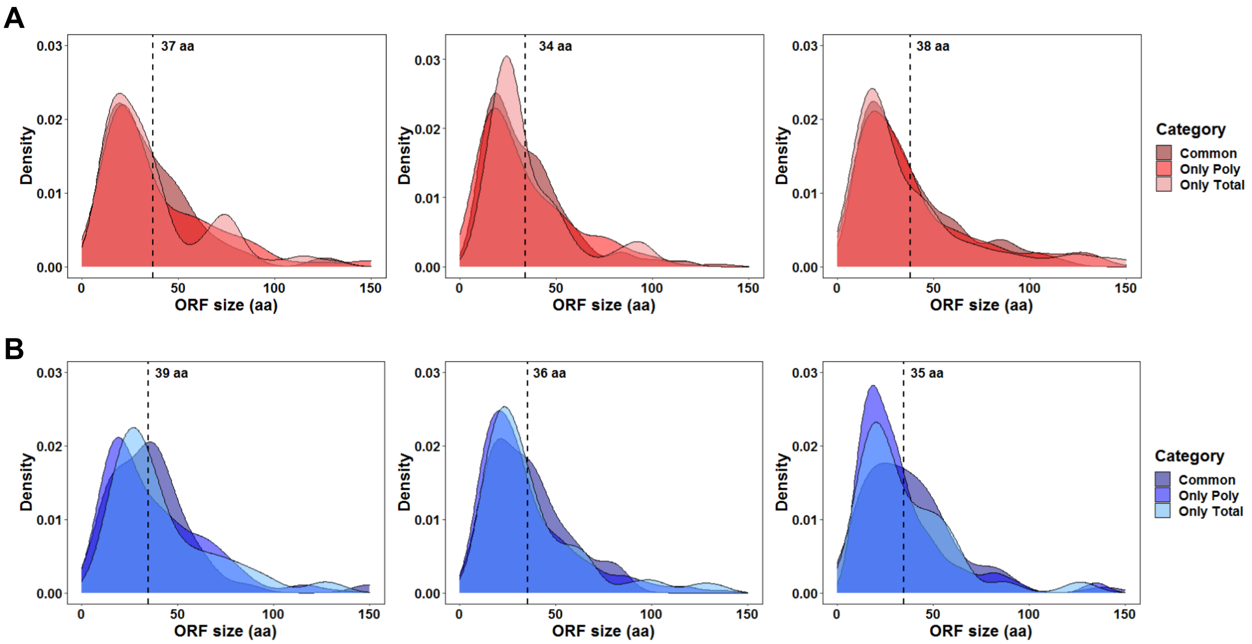

Figure S2: Density plot illustrating the size distribution of small open reading frames (smORFs) within differentially expressed lncRNAs during 24 hours of adipogenesis and osteogenesis. (A) The panel displays the size (aa) distribution of smORFs identified in frame 1, 2 and 3 of lncRNAs expressed during adipogenesis, respectively. **(B)** The panel shows the size (aa) distribution of smORFs identified in the frame 1, 2 and 3 of lncRNAs expressed during osteogenesis, respectively. The categories include: "Common", representing lncRNAs expressed in both the total and polysomal fraction; "Only Total", representing lncRNAs exclusively expressed in the total fraction; and "Only Poly", representing lncRNAs exclusively expressed in the polysomal fraction.

Supplementary Figure 3

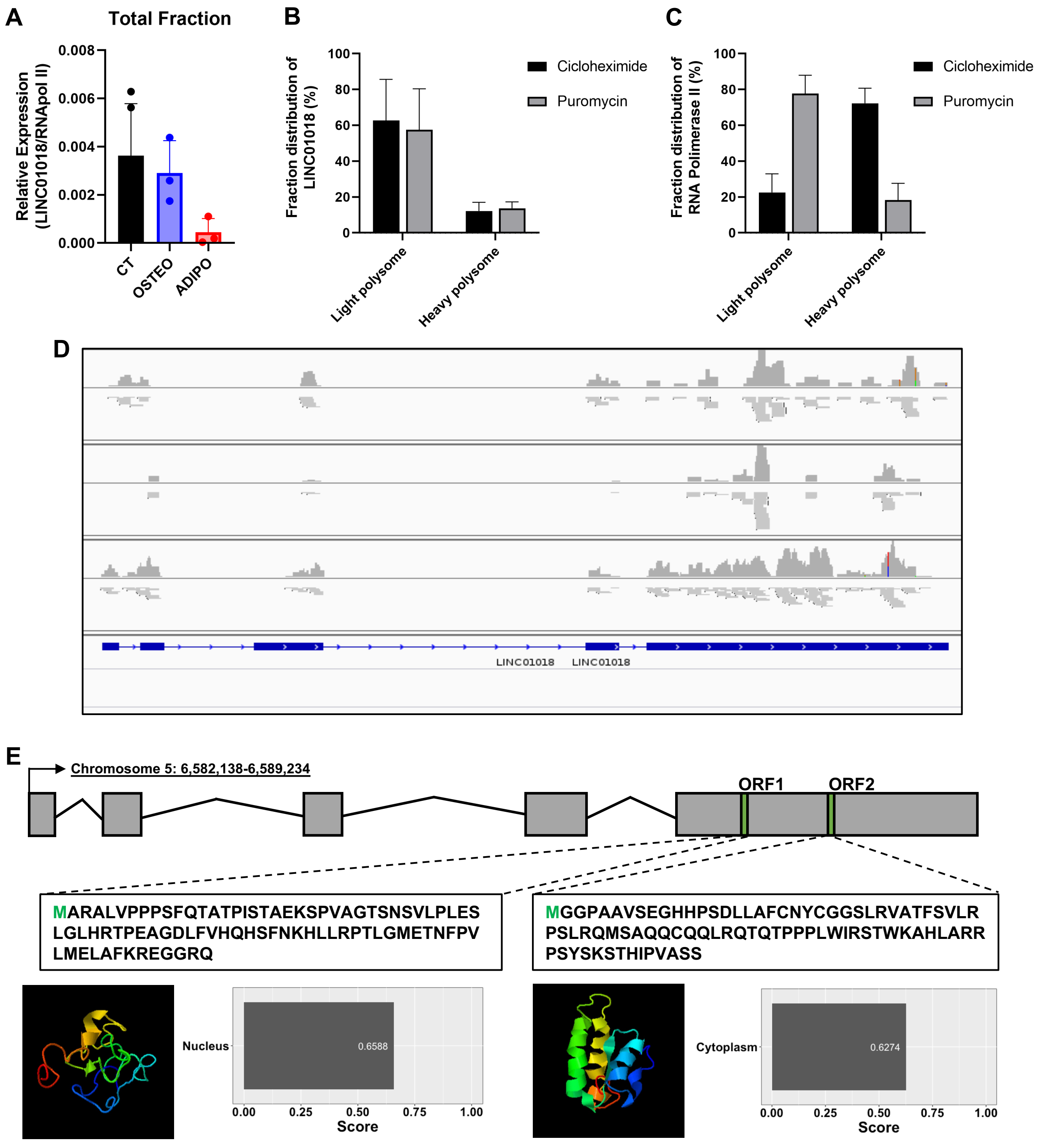

Figure S3: Polysome-associated LINC01018. (A) Expression levels of the polysomal-exclusive lncRNA LINC01018 as determined by RT-qPCR in the total fraction (n=3). **(B)** Expression levels of LINC01018 in the light polysome and heavy polysome fractions with cycloheximide or puromycin treatment. **(C)** Expression levels of POLR2A in the light polysome and heavy polysome fractions with cycloheximide or puromycin treatment. **(D)** Polysomal expression

coverage of the LINC01018 in three biological replicates after 24 hours of osteogenic differentiation. **(E)** Schematic representation of the LINC01018 transcript isoform and putative predicted ORF1 and ORF2 peptide sequence, along with structure and location predictions. Predicted 3D structures were generated using I-Tasser, and locations were predicted using DeepLoc 2.0.

Supplementary Table 1: Sequence of the oligonucleotides

Gene name	Sequence 5’-3’
**POLR2A-F**<br>**POLR2A -R**	TACCACGTCATCTCCTTTGATGGCT<br>GTGCGGCTGCTTCCATAA
**GFP-F**<br>**GFP-R**	TGAAGTTCGAGGGCGACACC<br>GATGTTGCCGTCCTCCTTGAAGT
**LINC01018-F**<br>**LINC01018-R**	CTGAGGCCAACACTGGGAAT<br>GGAAAGTCCGAGACCACGTT

Supplementary Table 2: Differentially expressed lncRNAs after 24 h of adipogenesis (Total Fraction).

Ensembl ID	lncRNA	Log2FC	FDR
ENSG00000224260	RP1-272L16.1	8.95536389	5.68E-13
ENSG00000230695	AC012462.1	7.94710923	3.10E-07
ENSG00000267480	RP11-703I16.1	7.77883512	1.54E-05
ENSG00000249609	RP11-635L1.3	7.53946248	5.60E-05
ENSG00000224251	RP11-499O7.7	7.19036065	0.00012292
ENSG00000226604	PAPPA-AS2	6.96186343	0.00049721
ENSG00000254860	TMEM9B-AS1	5.75160772	2.17E-06
ENSG00000196972	LINC00087	5.42839945	0.00026015
ENSG00000261578	RP11-21L23.2	5.20346141	0.00013519
ENSG00000234362	AC104654.2	5.03622344	7.40E-11
ENSG00000236882	LINC01554	4.77679428	0.00034994
ENSG00000270640	RP11-373D23.2	4.52364266	0.00955651
ENSG00000225937	PCA3	4.1148481	0.00261037
ENSG00000278309	RP11-102K13.5	3.89240381	0.00224357
ENSG00000266904	LINC00663	3.65299324	0.00043746
ENSG00000256006	AC084117.3	3.49933219	0.00675332
ENSG00000262601	CTC-786C10.1	3.31874799	0.00045579
ENSG00000249641	HOXC13-AS	3.23103022	0.00021886
ENSG00000254622	NAV2-AS4	3.13807662	0.00321177
ENSG00000267882	RP4-569M23.5	2.93456244	0.00021278
ENSG00000272356	RP5-1112D6.8	2.88956525	0.00400075
ENSG00000261324	RP11-174G6.5	2.87932702	3.74E-08
ENSG00000255043	NAV2-AS5	2.87881796	1.04E-06
ENSG00000260630	SNAI3-AS1	2.80504131	0.00046042
ENSG00000257151	PWAR6	2.7697352	0.0002923
ENSG00000182165	TP53TG1	2.596586	0.00238894
ENSG00000272841	RP3-428L16.2	2.53248067	4.31E-12
ENSG00000245812	RP11-175K6.1	2.50009609	0.00962866
ENSG00000246430	LINC00968	2.28568133	1.15E-11
ENSG00000263934	SNORD3A	2.16100919	0.00495642
ENSG00000244879	GABPB1-AS1	2.0929611	3.39E-16
ENSG00000223891	OSER1-AS1	2.07381264	0.00326331
ENSG00000226950	DANCR	2.0415139	7.99E-09
ENSG00000234608	MAPKAPK5-AS1	1.9726833	1.66E-07
ENSG00000203709	C1orf132	1.96052779	0.00344312
ENSG00000237036	ZEB1-AS1	1.90067999	0.0011625
ENSG00000270022	RNU12	1.89928836	0.00029996
ENSG00000258096	RP11-474P2.2	1.87081358	0.00093803

ENSG00000250548	RP11-47I22.2	1.86276471	0.00937228
ENSG00000221949	LINC01465	1.85821905	0.00216832
ENSG00000254343	RP11-760H22.2	1.79817799	0.00711256
ENSG00000280339	RP11-736K20.4	1.74980542	0.00206087
ENSG00000255471	RP11-736K20.5	1.54273482	0.00492093
ENSG00000262074	SNORD3B-2	1.54204383	2.07E-07
ENSG00000233117	LINC00702	-1.7359449	0.00556066
ENSG00000172965	MIR4435-1HG	-1.7804279	1.24E-08
ENSG00000269825	CTD-3099C6.9	-2.1398158	0.00075966
ENSG00000203706	SERTAD4-AS1	-2.6051121	0.00070679
ENSG00000231290	APCDD1L-AS1	-2.6501827	1.67E-07
ENSG00000280206	CTB-193M12.5	-2.723787	0.00030036
ENSG00000279338	RP1-309I22.2	-3.2302958	1.59E-22
ENSG00000239332	LINC01119	-3.2642909	0.00099331
ENSG00000248698	LINC01085	-3.4051041	9.85E-06
ENSG00000226876	RP11-36N20.1	-3.6066941	0.00972605
ENSG00000261760	RP11-1223D19.1	-3.6195576	1.89E-06
ENSG00000230417	LINC00856	-3.7832421	0.00053757
ENSG00000229563	LINC01204	-3.8778451	0.00218225
ENSG00000246763	RGMB-AS1	-3.9306774	3.35E-09
ENSG00000272327	RP11-1002K11.1	-4.3408644	1.30E-09
ENSG00000250064	RP11-123O22.1	-5.9467682	3.81E-08

Supplementary Table 3: Differentially expressed lncRNAs after 24 h of adipogenesis (Polysomal Fraction).

Ensembl ID	lncRNA	Log2FC	FDR
ENSG00000253716	RP13-582O9.5	7.530705153	2.70E-07
ENSG00000224260	RP1-272L16.1	5.951644544	1.57E-13
ENSG00000234362	AC104654.2	4.646466402	1.25E-18
ENSG00000267280	TBX2-AS1	4.23752777	0.00093431
ENSG00000261578	RP11-21L23.2	4.108329289	0.00079322
ENSG00000214900	C14orf182	4.020996702	2.85E-09
ENSG00000266921	RP11-15A1.7	3.851683314	0.00164687
ENSG00000266088	RP5-1028K7.2	3.83623786	0.00031534
ENSG00000267480	RP11-703I16.1	3.725218469	0.00081168
ENSG00000236882	LINC01554	3.635685843	1.66E-06
ENSG00000230838	AC093850.2	3.538565494	0.00015827
ENSG00000249641	HOXC13-AS	3.098619881	4.48E-06
ENSG00000259863	SH3RF3-AS1	3.097397489	2.41E-06
ENSG00000247095	MIR210HG	2.971122156	0.00713805
ENSG00000280339	RP11-736K20.4	2.80192607	5.12E-05
ENSG00000255471	RP11-736K20.5	2.755211097	4.23E-06
ENSG00000255284	AP006621.5	2.687673129	0.00177963
ENSG00000234779	RP11-62F24.2	2.661073412	5.25E-05
ENSG00000244586	WNT5A-AS1	2.41993777	0.00051081
ENSG00000267254	ZNF790-AS1	2.306293369	0.00232235
ENSG00000247271	ZBED5-AS1	2.293251778	0.00018394
ENSG00000272841	RP3-428L16.2	2.28002333	1.09E-14
ENSG00000244879	GABPB1-AS1	2.279768789	1.01E-12
ENSG00000261069	SNORD116-20	2.260101871	0.0024511
ENSG00000228035	RP4-663N10.1	2.172479825	0.00196921
ENSG00000182165	TP53TG1	1.944210954	0.00022114
ENSG00000223478	RP11-545E17.3	1.921926846	0.00123218
ENSG00000228630	HOTAIR	1.914560232	0.00801433
ENSG00000246430	LINC00968	1.892859691	1.66E-09
ENSG00000226950	DANCR	1.746807871	2.66E-09
ENSG00000267858	MZF1-AS1	1.744419028	0.00058166
ENSG00000237037	NDUFA6-AS1	1.713869561	0.0074247
ENSG00000223764	RP11-54O7.3	1.667916745	3.93E-07
ENSG00000245812	RP11-175K6.1	1.577973203	0.00745688
ENSG00000233016	SNHG7	1.502630843	5.98E-09
ENSG00000261105	LMO7-AS1	-1.542920484	0.00427174
ENSG00000261801	LOXL1-AS1	-1.72056126	0.00011896
ENSG00000261888	AC144831.1	-1.756336808	0.00227249

ENSG00000234840	LINC01239	-1.919795821	0.00550637
ENSG00000245146	LINC01024	-1.939487163	0.00095796
ENSG00000172965	MIR4435-1HG	-1.993752657	1.97E-16
ENSG00000279338	RP1-309I22.2	-2.053941369	0.00113715
ENSG00000231290	APCDD1L-AS1	-2.133839962	2.69E-11
ENSG00000203706	SERTAD4-AS1	-2.172680021	4.42E-05
ENSG00000270069	MIR222HG	-2.222493747	5.35E-08
ENSG00000253658	RP11-600K15.1	-2.225066902	8.99E-05
ENSG00000231607	DLEU2	-2.261792544	2.82E-05
ENSG00000257167	TMPO-AS1	-2.263799585	0.00024767
ENSG00000269825	CTD-3099C6.9	-2.266672232	6.10E-06
ENSG00000239332	LINC01119	-2.278719327	8.30E-06
ENSG00000226380	MIR29B1	-2.301209422	0.00281846
ENSG00000233117	LINC00702	-2.302102305	1.45E-11
ENSG00000267325	LINC01415	-2.308215852	4.19E-07
ENSG00000272341	RP1-151F17.2	-2.563655137	0.0033292
ENSG00000246763	RGMB-AS1	-2.794201724	0.00638142
ENSG00000248698	LINC01085	-2.931921964	9.19E-14
ENSG00000280206	CTB-193M12.5	-2.93393814	1.95E-05
ENSG00000272327	RP11-1002K11.1	-2.962643524	7.01E-09
ENSG00000236255	AC009404.2	-2.985463416	4.74E-07
ENSG00000250056	LINC01018	-3.060046509	0.00070903
ENSG00000233521	RP5-1172A22.1	-3.117578013	0.00119474
ENSG00000267577	CTD-2587H24.5	-3.218376631	0.00077641
ENSG00000241288	RP11-379B18.5	-3.255478242	0.00098444
ENSG00000230417	LINC00856	-3.316000555	8.09E-08
ENSG00000250303	RP11-356J5.12	-3.338218711	1.51E-05
ENSG00000223811	RP11-437J19.1	-3.413961478	0.00011181
ENSG00000229563	LINC01204	-3.452106553	1.58E-09
ENSG00000261760	RP11-1223D19.1	-3.54444315	3.10E-13
ENSG00000272975	MYHAS	-3.861259734	2.10E-06
ENSG00000248869	RP11-138I17.1	-3.90458642	2.82E-07
ENSG00000253161	RP11-150O12.1	-5.832715224	1.16E-09
ENSG00000250038	RP11-180C1.1	-5.963250704	6.25E-18

Supplementary Table 4: Differentially expressed lncRNAs after 24 h of osteogenesis (Total Fraction).

Ensembl ID	lncRNA	Log2FC	FDR
ENSG00000224260	RP1-272L16.1	8.27274105	1.16E-06
ENSG00000261386	CTD-2012K14.6	7.87016063	2.50E-05
ENSG00000249456	RP11-298J20.4	7.28298159	0.00039033
ENSG00000267480	RP11-703I16.1	7.08731748	0.00407812
ENSG00000249609	RP11-635L1.3	6.53922244	0.00582529
ENSG00000268894	PLCE1-AS1	6.42532621	5.80E-31
ENSG00000230838	AC093850.2	5.10323829	1.11E-05
ENSG00000261578	RP11-21L23.2	5.02110855	0.00934621
ENSG00000236882	LINC01554	4.8419407	0.00092097
ENSG00000259828	RP11-63E9.1	4.81422532	0.00519973
ENSG00000266088	RP5-1028K7.2	4.68538911	0.00339928
ENSG00000254987	RP11-563P16.1	3.90095	0.00019709
ENSG00000235770	LINC00607	3.72552109	1.16E-20
ENSG00000237697	LINC00312	3.65672434	6.89E-05
ENSG00000278133	RP11-196G11.5	3.22191282	0.00198115
ENSG00000248890	HHIP-AS1	3.15306905	0.00803823
ENSG00000197301	RP11-366L20.2	2.83167569	1.75E-06
ENSG00000254343	RP11-760H22.2	2.77444352	2.62E-06
ENSG00000261324	RP11-174G6.5	2.38709224	0.00205279
ENSG00000255043	NAV2-AS5	2.16566294	0.009629
ENSG00000225135	RP11-361F15.2	1.93628009	3.60E-05
ENSG00000259330	INAFM2	1.89222047	9.10E-06
ENSG00000237187	NR2F1-AS1	1.61458413	0.00081016
ENSG00000246430	LINC00968	1.54349814	0.00255898
ENSG00000235531	RP11-383H13.1	-1.6689233	6.54E-06
ENSG00000231290	APCDD1L-AS1	-1.8100121	0.00048887
ENSG00000279095	AC092066.1	-1.9891353	7.76E-05
ENSG00000224259	LINC01133	-2.0264139	4.93E-12
ENSG00000249378	LINC01060	-2.1385667	0.00257648
ENSG00000224743	TEX26-AS1	-2.8180509	0.00350366
ENSG00000236255	AC009404.2	-3.0046471	0.00024527
ENSG00000223749	MIR503HG	-3.0706649	1.04E-10
ENSG00000272327	RP11-1002K11.1	-3.6161858	9.04E-09
ENSG00000273002	RP11-336K24.12	-3.8381108	0.00158531
ENSG00000226876	RP11-36N20.1	-4.1197155	0.00238335
ENSG00000250064	RP11-123O22.1	-4.967432	6.17E-06
ENSG00000250038	RP11-180C1.1	-5.4708315	0.0037106

Supplementary Table 5: Differentially expressed lncRNAs after 24 h of osteogenesis (Polysomal Fraction).

Ensembl ID	lncRNA	Log2FC	FDR
ENSG00000224260	RP1-272L16.1	5.61446932	1.81E-07
ENSG00000247311	CTC-441N14.2	5.24496134	1.30E-07
ENSG00000239828	RP11-446H18.5	4.83652092	8.53E-05
ENSG00000266088	RP5-1028K7.2	4.73969229	1.63E-06
ENSG00000268894	PLCE1-AS1	4.70673837	2.61E-12
ENSG00000225511	LINC00475	4.61525614	0.00127184
ENSG00000267080	ASB16-AS1	4.36118524	0.00175614
ENSG00000237697	LINC00312	4.21396142	6.92E-05
ENSG00000272053	RP11-367G6.3	4.20142509	0.00478782
ENSG00000276075	CTD-2012K14.8	4.1767546	0.00212674
ENSG00000267480	RP11-703I16.1	4.15679601	0.00022178
ENSG00000235770	LINC00607	3.77380221	3.25E-19
ENSG00000274627	RP11-104N10.2	3.65309568	0.00805376
ENSG00000254510	RP11-867G23.10	3.63900132	0.00627922
ENSG00000249641	HOXC13-AS	3.46338392	1.82E-05
ENSG00000254987	RP11-563P16.1	3.38765673	3.45E-08
ENSG00000236882	LINC01554	3.38477428	3.00E-05
ENSG00000279204	RP11-175K6.2	3.34309396	0.00348106
ENSG00000280339	RP11-736K20.4	3.22222341	9.50E-05
ENSG00000255471	RP11-736K20.5	2.85508205	0.00041961
ENSG00000259450	RP11-265N7.1	2.66356503	1.22E-05
ENSG00000197301	RP11-366L20.2	2.62418661	8.11E-08
ENSG00000236671	PRKG1-AS1	2.62223657	0.00370475
ENSG00000227145	IL21-AS1	2.53099605	0.00055719
ENSG00000268913	AC026806.2	2.4952266	0.00725821
ENSG00000245532	NEAT1	2.41328672	8.35E-09
ENSG00000259330	INAFM2	2.40575516	2.73E-10
ENSG00000260910	LINC00565	2.37965217	0.00048223
ENSG00000228035	RP4-663N10.1	2.32457812	0.00637134
ENSG00000269990	CTD-3074O7.12	2.24447762	0.00354302
ENSG00000245812	RP11-175K6.1	2.19603231	0.00125471
ENSG00000229729	RP11-159G9.5	2.09690741	0.001754
ENSG00000254343	RP11-760H22.2	2.01373481	0.00342695
ENSG00000248890	HHIP-AS1	2.00875631	0.00476525
ENSG00000182165	TP53TG1	1.92120378	0.0074896
ENSG00000279118	RP11-517I3.2	1.86168046	0.00018131
ENSG00000280143	AP000892.6	1.81484249	9.70E-05

ENSG00000240859	AC093627.10	1.73396622	0.00035401
ENSG00000250056	LINC01018	1.7111585	0.00296366
ENSG00000272565	RP11-485G4.2	1.64333005	0.00056965
ENSG00000242539	AC007620.3	1.63676392	0.00015216
ENSG00000272695	GAS6-AS2	1.60099883	0.00799659
ENSG00000246430	LINC00968	1.55378212	0.00733645
ENSG00000272933	RP11-47A8.5	1.5530872	0.00581199
ENSG00000226950	DANCR	1.54120048	0.00138728
ENSG00000248187	RP11-184M15.1	-1.5100959	0.00094564
ENSG00000261040	WFDC21P	-1.5785988	0.00091492
ENSG00000269893	SNHG8	-1.5794887	2.98E-05
ENSG00000232774	FLJ22447	-1.6067996	0.00547754
ENSG00000235531	RP11-383H13.1	-1.6282531	5.80E-05
ENSG00000224032	EPB41L4A-AS1	-1.6840356	0.00020663
ENSG00000177410	ZFAS1	-1.6942858	2.81E-06
ENSG00000234741	GAS5	-1.7054639	0.00012727
ENSG00000261824	LINC00662	-1.8985823	5.82E-05
ENSG00000249378	LINC01060	-1.9636872	0.00455344
ENSG00000224259	LINC01133	-2.3656047	4.10E-11
ENSG00000272975	MYHAS	-2.4100178	0.00604814
ENSG00000272327	RP11-1002K11.1	-2.6186856	0.00010566
ENSG00000230623	RP11-469A15.2	-3.3121347	0.00088262
ENSG00000236404	VLDLR-AS1	-3.547759	0.00064381
ENSG00000235513	RP4-756G23.5	-3.8284574	0.00038099
ENSG00000272341	RP1-151F17.2	-3.8913966	0.00182014
ENSG00000223749	MIR503HG	-4.055987	0.00037501
ENSG00000231196	RP11-495P10.8	-6.1044841	1.79E-06
ENSG00000250038	RP11-180C1.1	-8.1864425	6.90E-18
